# Metastatic Anal Fistula Carcinoma after Rectosigmoid Cancer Surgery Treated with Robot-Assisted Abdominoperineal Resection and Gluteal Myocutaneous Flap Reconstruction: A Case Report

**DOI:** 10.70352/scrj.cr.26-0108

**Published:** 2026-06-12

**Authors:** Aina Kunitomo, Tatsuki Matsumura, Kentaro Shinohara, Yasuyuki Fukami, Kenitiro Kaneko, Shunichiro Komatsu, Emiko Takahashi, Toyonori Tsuzuki, Tsuyoshi Sano

**Affiliations:** 1 Department of Gastroenterological Surgery, Aichi Medical University Hospital, Nagakute, Aichi, Japan; 2 Department of Surgery, Tokai Hospital, Nagoya, Aichi, Japan; 3 Department of Surgical Pathology, Aichi Medical University Hospital, Nagakute, Aichi, Japan

**Keywords:** metastatic anal fistula carcinoma, robot-assisted laparoscopic abdominoperineal resection, gluteal myocutaneous flap

## Abstract

**INTRODUCTION:**

Robot-assisted surgery has become an established approach for rectal cancer, offering favorable short-term outcomes and oncologic results comparable to conventional laparoscopic surgery. With recent technological advances, its use has expanded to more complex scenarios, including reoperative surgery with severe adhesions or distorted pelvic anatomy. Metastatic anal fistula carcinoma is uncommon, and its surgical management is challenging, particularly in patients with prior rectal surgery because of severe adhesions and distorted pelvic anatomy. We report a rare case of metastatic carcinoma associated with an anal fistula that developed after curative resection of rectosigmoid cancer and was treated with robot-assisted abdominoperineal resection (R-APR) and gluteal myocutaneous flap reconstruction.

**CASE PRESENTATION:**

A 61-year-old man who had previously undergone laparoscopic high anterior resection for Stage IIIB rectosigmoid cancer presented 13 months later with anal pain and swelling. Imaging revealed a mass connected to the rectum through a chronic fistulous tract, and biopsy confirmed adenocarcinoma. A primary carcinoma arising in an anal fistula or local recurrence of rectosigmoid cancer was considered. R-APR was performed using the da Vinci Xi system. Severe adhesions around the prior anastomosis were meticulously dissected, allowing safe total mesorectal excision and resection of the tumor and fistula tract. The large perineal defect was reconstructed with bilateral gluteal myocutaneous flaps. No intraoperative complications occurred. Histopathological examination showed an adenocarcinoma that was morphologically identical to the previously resected rectosigmoid cancer with no evidence of associated in situ lesions. In addition, both tumors demonstrated CK7 (−)/CK20 (+) staining on immunohistochemical examination, which is most consistent with metastatic anal fistula carcinoma. Surgical margins were negative, and no recurrence was observed during 12 months of follow-up.

**CONCLUSIONS:**

This case suggests that the robotic platform may facilitate safe dissection in complex reoperative pelvic procedures. Gluteal myocutaneous flap reconstruction provided effective coverage for a large perineal defect in this patient.

## Abbreviation

R-APR
: robot-assisted laparoscopic abdominoperineal resection

## INTRODUCTION

Robot-assisted surgery has become a standard approach for rectal cancer, offering favorable short-term outcomes and oncologic results comparable to those of conventional laparoscopic surgery.^1,2)^ Recent technological advances have broadened its application beyond routine rectal cancer resections to more complex scenarios, including patients with extensive adhesions or distorted pelvic anatomy due to prior surgery or chronic inflammatory conditions such as Crohn’s disease–associated carcinoma.^3)^

Anal fistula carcinoma is a rare entity, and metastatic cases are even more uncommon, with only a limited number of case reports available in the literature. Surgical management is technically challenging, particularly in patients with prior rectal surgery, where dense adhesions and distorted pelvic anatomy are often present. Here, we report a case of metastatic anal fistula carcinoma that developed after curative resection of rectosigmoid cancer and was treated with R-APR followed by bilateral gluteal myocutaneous flap reconstruction. This case illustrates the feasibility of robot-assisted abdominoperineal resection in a reoperative pelvis with severe adhesions and highlights the role of myocutaneous flap reconstruction in managing large perineal defects.

## CASE PRESENTATION

A 61-year-old man had previously undergone laparoscopic high anterior resection with D2 lymphadenectomy for rectosigmoid colon cancer. Histopathological examination revealed an adenocarcinoma staged as pT3N1aM0 (Stage IIIB, Tumor Node Metastasis 8th edition). Tumor size was 5.0 × 4.0 cm, and vascular invasion was observed, but no lymphatic invasion was detected, which was confirmed by D2-40 immunostaining. Proximal, distal, and radial surgical margins were negative. As the patient was on maintenance hemodialysis for diabetic nephropathy, postoperative adjuvant chemotherapy was not administered.

Thirteen months after surgery, the patient presented to our hospital with anal pain and perianal swelling. In addition to diabetic nephropathy, his medical history included a long-standing anal fistula (>20 years) and chronic atrial fibrillation. Maintenance dialysis was performed 3 times a week. The patient had undergone ablation therapy for chronic atrial fibrillation and was not receiving anticoagulant therapy.

**Fig. 1:**
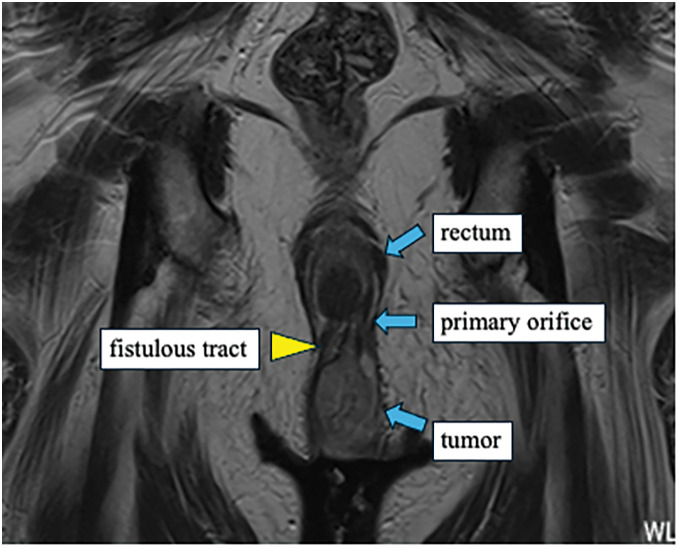
Pelvic MRI demonstrating a 56 × 21-mm tumor connected to the rectum via a fistulous tract (arrowhead).

Serum carcinoembryonic antigen and carbohydrate antigen 19-9 levels were 6.7 ng/mL and 12 U/mL, respectively. Abdominal CT and pelvic MRI revealed a well-defined mass (56 × 21 mm) in the left buttock adjacent to the anus, contiguous with the rectum via a fistulous tract (**Fig. 1**). Colonoscopy showed no abnormalities at the anastomotic site (15 cm from the anal verge) and no obvious fistula within the anal canal. Retrospective review of the preoperative CT images obtained at the time of the initial rectal cancer surgery confirmed the presence of an anal fistula but revealed no tumorous lesion. Furthermore, during the initial operation, the anal canal was carefully inspected at the time of double-stapling anastomosis, and no abnormal or tumorous findings were noted. Subsequently, drainage of a perianal abscess and biopsy of the newly developed mass demonstrated adenocarcinoma. Based on these findings, a differential diagnosis of primary carcinoma arising in a chronic anal fistula versus metastatic recurrence of rectosigmoid cancer within the anal fistula was considered.

The patient underwent R-APR using the da Vinci Xi Surgical System (Intuitive Surgical, Sunnyvale, CA, USA). The procedure included resection of the fistulous tract and the perianal mass with the patient in the lithotomy position, followed by bilateral gluteal myocutaneous flap reconstruction to repair the perineal defect.

**Fig. 2:**
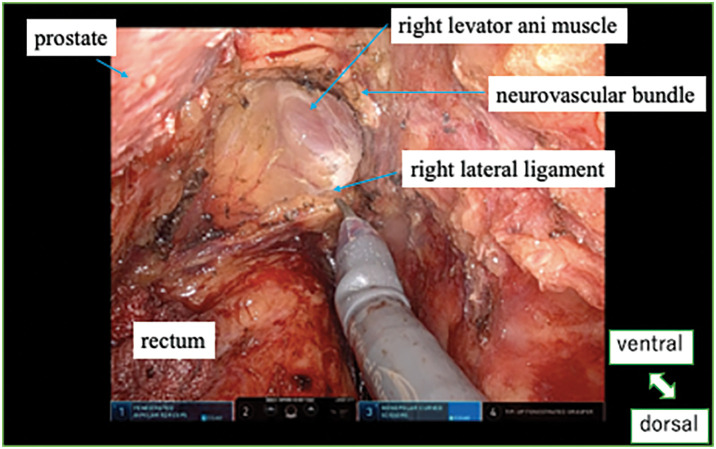
Robot-assisted rectal mobilization demonstrating safe dissection toward the pelvic floor with a clear operative view.

Four 8-mm robotic trocars were placed in a straight line from the right lower abdomen to the left upper abdomen for the robotic instruments and camera, with an additional 12 and 5-mm trocar for the assistant. Despite severe adhesions resulting from the prior surgery, the descending colon was carefully mobilized using a medial approach. As the mesentery could be dissected distal to the previous superior rectal artery resection margin, no additional vascular ligation was required. Rectal mobilization was performed with traction on the descending colon using gauze, in accordance with standard principles of rectal cancer surgery. The enhanced 3D visualization and articulated instrument control of the robotic system facilitated meticulous dissection of dense adhesions around the previous anastomosis, enabling safe total mesorectal excision and rectal mobilization to the pelvic floor (**Fig. 2**). The proximal colon was then divided, completing the robotic phase of the procedure.

**Fig. 3:**
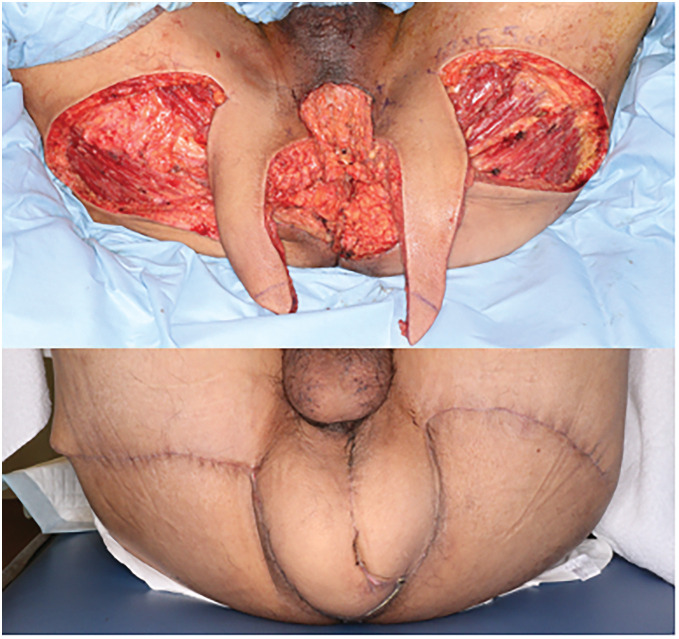
Bilateral gluteal myocutaneous flap reconstruction providing secure coverage of the perineal defect.

Perineal dissection was performed next, including en bloc resection of the tumor and fistula tract, with transperineal specimen extraction. The plastic surgery team reconstructed the 12 × 12-cm perineal defect using bilateral 12 × 6.5-cm gluteal myocutaneous transposition flaps (**Fig. 3**). Adequate flap perfusion was confirmed with indocyanine green before closure. Subcutaneous drains were placed bilaterally, and a pelvic drain was inserted laparoscopically. Finally, a descending colostomy was created. The total operative time was 14 h 20 min, with an estimated blood loss of 553 mL. Operative time was approximately 6 h for the abdominal phase, about 1 h for the perineal phase, 5 h and 50 min for the reconstructive phase, and about 40 min for wound closure and stoma creation phase. The prolonged operative time of the abdominal phase was mainly due to severe adhesions.

**Fig. 4:**
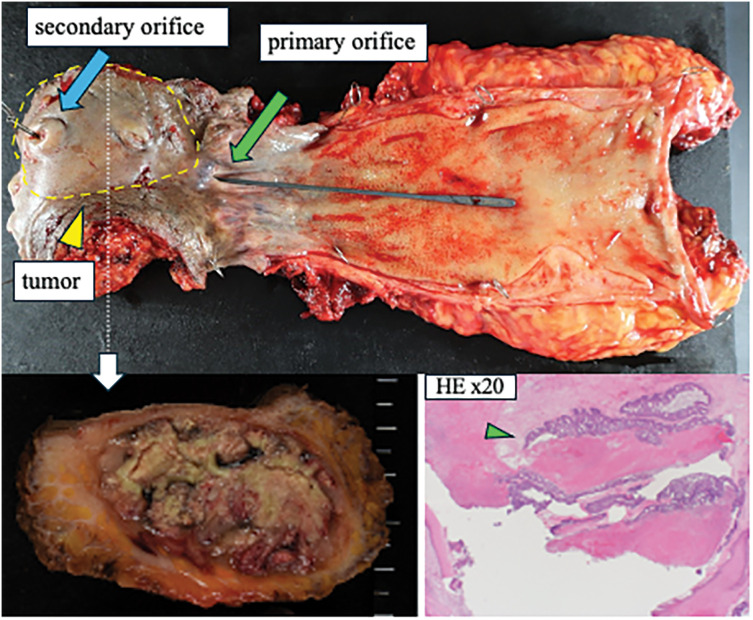
Macroscopic findings showing the primary orifice (green arrow) communicating with the secondary orifice (blue arrow) via a fistulous tract within the tumor (yellow dotted circle). Histopathological findings demonstrate an adenocarcinoma located beneath intact squamous epithelium, without continuity with the overlying epithelium (arrowhead). H&E, hematoxylin and eosin

**Fig. 5:**
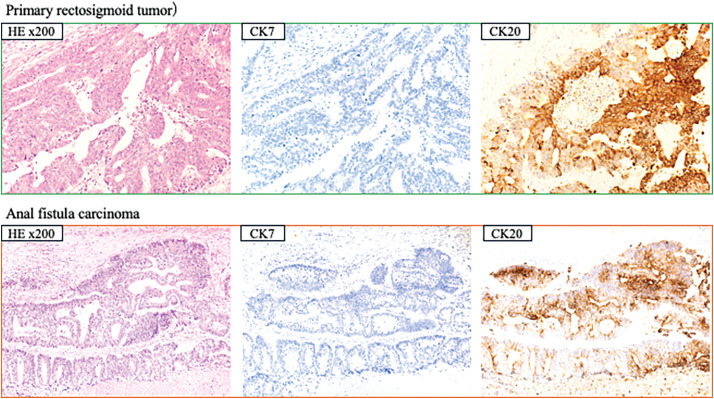
Histopathological and immunohistochemical comparison between the primary rectosigmoid tumor (upper panels) and the anal fistula carcinoma (lower panels). Both lesions demonstrate similar morphological features on hematoxylin and eosin staining and share an identical immunophenotype of CK7 (−)/CK20 (+), supporting a colorectal origin. H&E, hematoxylin and eosin

Macroscopically, the tumor size measured 6.0 × 5.0 cm. The primary fistula orifice was located at the dentate line and communicated with the tumor through a fistulous tract. Gross examination of the specimens, including serial sectioning, revealed no anal glands within the fistula tract. Histopathological examination demonstrated intact squamous epithelium lining the fistula tract, with the tumor located predominantly in the subepithelial interstitium and showing no continuity with the overlying epithelium (**Fig. 4**). The resected specimen showed no metastatic lymph nodes. Proximal, distal, and radial surgical margins were negative. The tumor was composed of an adenocarcinoma with morphological features identical to those of the previously resected rectal cancer in hematoxylin and eosin stains. In addition, CK7/CK20 immunohistochemical examination was performed on both the primary tumor and the anal fistula carcinoma, both of which showed CK7 negativity and CK20 positivity (CK7 (−)/CK20 (+)) (**Fig. 5**). Based on the histological concordance with the prior rectal cancer, the absence of any in situ lesion, and the immunohistochemical profile (CK7 (−)/CK20 (+)), the lesion was most consistent with metastatic anal fistula carcinoma originating from the prior rectosigmoid carcinoma. Resection margins were negative. The postoperative course was uneventful, and maintenance hemodialysis was performed according to the regular schedule. The patient was discharged on POD 17. At 12 months of follow-up, no evidence of recurrence was observed.

## DISCUSSION

We report a rare case of metastatic anal fistula carcinoma managed with R-APR. In this patient, prior rectal surgery had resulted in severe adhesions and distorted pelvic anatomy, and robotic assistance enabled precise and safe dissection in a challenging reoperative field. Although each component of this case—including anal fistula carcinoma, R-APR, and myocutaneous flap reconstruction—has been individually reported, the present case is unique because it represents a rare combination of metastatic anal fistula carcinoma arising after rectosigmoid cancer surgery, managed in a reoperative pelvis using a robotic approach with simultaneous gluteal myocutaneous flap reconstruction.

Metastatic anal fistula carcinoma is an exceedingly rare condition, first described by Guiss.^4)^ It is believed to arise from the implantation of cancer cells migrating from advanced colon cancer into an anal fistula. In this case, the clinical course involved the development and rapid growth of the tumor within an anal fistula that showed no abnormalities whatsoever during rectal cancer surgery 1 year prior. Moreover, no continuity was observed macroscopically or microscopically between the tumor and the overlying epithelium. The primary rectal cancer lesion showed vascular invasion on histopathology, suggesting a potential for hematogenous dissemination. Therefore, implantation of exfoliated tumor cells into the fistula tract is considered a more plausible mechanism than lymphatic spread in this case. Based on these findings and the histopathological similarity between the prior rectal cancer and the anal fistula tumor, we considered the lesion most consistent with metastatic anal fistula carcinoma. Additionally, previous reports^5)^ indicate that immunostaining was useful for distinction between primary and metastatic anal fistula carcinoma. Loy and Calaluce^6)^ reported that the vast majority of colon cancers (80%) are CK7 (−)/CK20 (+), while some (16%) are CK7 (+)/CK20 (+) and a few (4%) are CK7(−)/CK20 (−). Hobbs et al.^7)^ found that 6 of 7 cases of primary cancer arising from an anal fistula were CK7(+)/CK20 (−). The specimen from this case showed CK7 (−)/CK20 (+), which supports the diagnosis of metastatic anal fistula carcinoma.

Complete resection of both the fistula and the tumor as curative treatment often requires extensive surgery. In metachronous cases, prior colorectal surgery further complicates the procedure, as adhesions and altered anatomy increase the risk of intraoperative injury. In the present case, the robotic platform was particularly advantageous during dissection around the previous anastomosis, where dense adhesions and distorted anatomy were encountered. The enhanced 3D visualization and articulated instruments enabled more precise dissection in the confined pelvic space, facilitating a safe total mesorectal excision and rectal mobilization. However, the prolonged operative time in this case reflects the complexity of the procedure and represents a limitation of this approach.

Another important aspect of this case is the use of myocutaneous flap reconstruction. Large perineal defects after APR are prone to complications such as wound dehiscence, infection, and dead space formation.^8)^ Several studies have reported extended APR with myocutaneous flap reconstruction for anal fistula cancer, using donor sites including the rectus abdominis and anterolateral thigh flaps.^8–10)^ In this case, a gluteal myocutaneous flap was selected because it provides adequate coverage with less tissue volume than procedures such as total pelvic exenteration, is technically simpler than rectus abdominis or thigh flaps, and carries a relatively lower risk of complications. Moreover, the flap is amenable to elevation in the lithotomy position without interfering with abdominal port placement, allowing a smooth combined approach.

## CONCLUSIONS

We report a rare case of metastatic anal fistula carcinoma treated with R-APR and gluteal myocutaneous flap reconstruction. This case suggests that robot-assisted abdominoperineal resection combined with tailored reconstructive strategies is feasible in selected patients with complex reoperative pelvic conditions. However, the findings are limited by the single-case nature and relatively short follow-up period.
